# Do we need an SBIRT program?

**DOI:** 10.1186/1940-0640-8-S1-A43

**Published:** 2013-09-04

**Authors:** Hugo López Pelayo, Mireia Vàzquez Vallejo, Antoni Gual Solé

**Affiliations:** 1 Hospital Clínic Barcelona, Addictions Unit, Psychiatry Department, Clinic Neurosciences Institute, Barcelona, Catalonia, Spain; 2 Hospital Clínic Barcelona, Psychiatry Department, Clinic Neurosciences Institute, Barcelona, Catalonia, Spain

## 

Drug use has an impact on physical and mental health. Quite often, patients attend accident and emergency (A&E) departments due to drug-use-related disorders, and this is why screening and brief intervention for substance use disorders are relevant in emergency room settings. Studies have shown that screening, brief intervention, and referral to treatment (SBIRT) programmes are effective in several medical settings, including A&E departments. Since most of the evidence has been gathered in the US, this study tries to identify the relevance of substance use disorders in a large Spanish A&E Department and the possible need to implement a SBIRT programme. All medical doctors on duty in a given day were asked prospectively about ward consultations attended during their 16 hours-shift. The questionnaire included information on patients’ substance use and if this was related to the medical visit, whether or not they thought the patient needed referral to an addiction specialist and their opinion on what may help them to improve the management of those patients. 194 patients were attended. 22 patients (11.3%) were reported as substance users. Only 2 were visited by the psychiatrist (1%). Alcohol (n=10), cannabis (n=3) and benzodiazepines (n=4) were the most reported drugs (n=10). Practitioners considered that support from an addiction specialist (8, 7/10) and specific training in the management of substance users (8, 2/10) as the most valuable aids. Availability of screening tools was somewhat less valued (7, 2/10). The number of patients who present to the A&E department with a drug related-problem is large, yet these patients are scarcely seen by the psychiatrist. Practitioners see training opportunities in the management of substance users and the availability of addiction specialists as the most valued aids. Regardless, the rate of patients presenting with substance use related disorders warrants further research and suggests the convenience to implement SBIRT programmes in A&E department settings.

